# Notes and News

**Published:** 1890-04-26

**Authors:** 


### NOTES AND NEWS.

Collected and Communicated.

In addition to the handsome legacy bequeathed by the late Sir James Tyler to the Merchant Taylors' Company for charitable purposes, the large sum of £42,000 was bequeathed to the London Missionary Society, and a similar sum to the British and Foreign Bible Society. The City of London Truss Society receives £2,000. These gifts are, however, subject to a life annuity.

The death is announced of Dr. George C. Jonson, of Eaton Place, one of the oldest and best supporters of Epsom College. For many years of his life Dr. Jonson was blind from cataract; but in spite of this disability he could make his way without assistance from his house to the Army and Navy Stores, and make his purchases as well as any one. He lived to have the cataract removed, and saw perfectly to the end of his life.

The Easter Fair in aid of the Western Skin Hospital, Great Portland Street, was a very bright and cheerful three days' festival, and we hope will be a substantial benefit to the worthy institution in whose aid it was organised. The tickets of admission were cheap, the entertainments were excellent, and the stalls tastefully arranged. One of the greatest draws was Professor Annie Oppenheim who undertook to read your character from your countenance. We wait with interest to hear the financial result of the Fair.

The Right Hon. the Earl of Meath will take the chair at the last meeting of the seventh session of the Hospitals Association on the 30th inst., at 8 p.m., at St. Thomas's Hospital. A paper will be read on the "New Street Ambulance Service of the Metropolis" by Mr. Thomas Ryan, Secretary to St. Mary's Hospital. We hear that among the members who have recently joined the Hospitals Association are:—Miss Stains, lady superintendent of the Liverpool Royal Infirmary and Nurses' Training School; Miss Yorke, matron, Macclesfield Infirmary, and the Rev. James Mahomed, chaplain of the London Hospital.

The serious sanitary defects of Derbyshire Infirmary necessitate immediate steps. Sixteen nurses have been ill with continued fever, and Dr. Seaton expresses his belief that the fever is due to the bad drainage. Then the question comes forward —a new hospital, or merely some patching? The more drastic remedy would be best, but as usual, there is the question of funds. In the meanwhile the squabble about the proper qualifications for the honorary medical officers must fall into abeyance, though Dr. Ogle has written to the committee saying he has brought the question before many people who mostly uphold his views, and that he has interviewed Miss Nightingale and Mr. Burdett on the subject.

The balance-sheet of the Nottingham General Hospital is a creditable one; it is clearly and fully drawn up, and tells a tale of reasonable economy; indeed, the cost per patient has been reduced from £5 11s. in 1883, to £4 13s. In-patients numbered 1,547, and out-patients 7,609 during last year. At the late annual meeting it was resolved to ask the Hospital Saturday Committee to nominate a workingman on the weekly board. On April 11th, through the efforts of Dr. Kingdom, the patients of No. 6 Ward in this hospital enjoyed an excellent concert. We are sure the patients at Nottingham are well cared for, and we congratulate the committee on having a matron who seems to take equal interest in the housekeeping and nursing departments.

The Bishop of Marlborough presided at the annual meeting of the Hospital for Epilepsy and Paralysis, when an excellent report was submitted. His Lordship, in the course of his speech, said that he had been permitted to make an announcement which, he was sure, all present would receive with the same pleasure as he felt in making it. It was to the effect that, through the munificence of an anonymous donor, who had promised a gift of over £800 for that express purpose, the purchase of the lease of Winterton House, so long the home of the hospital, had, with the help of the fund so energetically raised by Miss Althaus and her friends, become a possibility. This would save the hospital £130 a year for ten years, and would in other respects be of great advantage to the institution.

Visitors to the fasting man at the Aquarium, Succi, on Monday last, beheld a pale and emaciated being evidently in a state of nervousness and irritability. He sat in an armchair, his foot tapped the floor, or his fingers played on the table; he read part of the time, but he seldom spoke, and he paid little heed to the curious sight-seers. Succi had then entered on the 36th day of his fast, and hoped to succeed, though he was warned not to speak or move, but to husband every remnant of strength. The hollow cheeks, and still more the sunken eye-lids, told how far gone Succi was towards starvation. Not the least terrible part of this task must have been the perpetual presence of two of the watch committee; for forty days and nights, sleeping or waking, to be perpetually gazed on, never for a moment alone, must have been as great a trial to the nerves as the absence of food was to the body.

The festival dinner of the Hospital for Sick Children, Great Ormond Street, was held at the Hotel Metropole, on Tuesday evening, under the presidency of the Lord Mayor, when a large number of friends of the institution sat down to an excellent spread. The Lord Mayor, in proposing "Prosperity to the Hospital for Sick Children," said that this was the first hospital established in the United Kingdom specially devoted to the reception of sick children. It was established in the year 1852 with 20 beds, which number had been added to from time to time until at the present time it contained 127 beds. Besides this, the Convalescent Home at Highgate had 52 beds, making a total accommodation of 179 beds. As to the financial position of the institution, though there was no actual debt, yet the legacies, which averaged £2,000 a-year, were always swallowed up for current expenditure instead of being invested. There was only an income from investments of £400. The actual wants of the hospital now were (1) donations for present need and maintenance—at least £2,000; (2) new subscribers to the extent of £1,000 a-year; (3) a sum of £9,000 to complete the building which had been for so many years left unfinished. During the evening the secretary (Mr. Adrian Hope) announced that the subscriptions received at the dinner amounted to £3,000.

The following vacancies are announced this week, the amount of the salary in each case being given after the name of the institution :—

**Resident Medical Officers.**

Pontefract Dispensary, Medical Officer—£130, rooms, etc.
North-West London Hospital, House Physician—Board, etc.
Western General Dispensary, House Surgeon—£50, board, etc.
Shadwell Hospital for Children, House Physician—Board, etc.
Lancaster County Asylum, Pathologist—£200, board, etc.
Bolton Infirmary, Junior House Surgeon—£100, board, etc.
Edinburgh Poorhouse, Medical Officer—£80, board, etc.

**Non-Resident Medical Officers.**

Strathdon Parochial Board, Medical Officer—£65.
Metropolitan Hospital, Physician and Assistant Physician.
Owen's College, Senior Demonstrator in Physiology—£150.
Rangoon Municipality, Health Officer—Rs. 600 per mensem.
National Orthopædic Hospital, Surgical Registrar and Anæsthetist—£20.
Kirkpatrick, Medical Officer—£20.

---

The annual report of the Lancaster Infirmary is a carefully compiled pamphlet which ought to be useful to managers of other institutions for purposes of comparison. The table on page 15, giving the average cost per year of each occupied bed, is particularly excellent. The Resident Medical Officer's report is also full and complete, and the committee record their appreciation of his powers. Messrs. Paley and Austin will be the architects for the new infirmary.

---

The festival dinner in aid of the Royal Medical Benevolent College was attended by many distinguished medical men. Sir James Paget presided. The chairman having proposed the toasts of "The Queen and the Other Members of the Royal Family" and of "The Army and Navy," Dr. Hutchinson gave "The Houses of Parliament." Lord Granville replied in a jocular speech for the House of Lords. By some people, he said, the House of Lords was thought to be rather a sick patient, and it was a relief to him to find that the eminent physicians and surgeons present had not thought it necessary to propose any drastic remedy with regard to it. While in the reception-room he had asked a prominent physician what the doctors' patients would do that evening while they were all there dining together, and he was told that they would probably get better. This remark reminded him of a story he once heard of a man who contended that a cause might on occasion follow an effect. This was when a physician followed his patient to the grave. Sir Trevor Lawrence responded for the Commons. Sir James Paget afterwards proposed "Prosperity to the College," which was responded to by Dr. Holman, the treasurer. Subscriptions amounting to some £2,100 were announced.

---

Viscount Emlyn presided at the forty-third annual meeting of the governors of Carmarthenshire Infirmary. The House Committee reported that the Infirmary continued to be in a condition of complete efficiency. There was an increase in the subscriptions and donations, and in the amount collected in the churches and chapels during the past year; still, there was an adverse balance of £69 15s. 8d. This was almost entirely due to the large sums expended upon the necessary repairs of the institution. A sub-committee had made a searching inquiry into the expenditure, and proved that the institution had never been so economically managed as it had been under the direction of Miss Macintyre, whom the committee took an opportunity of publicly thanking for her valued services. There was ample room for many more in-patients, but it could not be afforded, since lack of funds had prevented the House Committee from furnishing the wards which stood empty, and if they were furnished, the income was too small to permit of an increased number of inmates. Gallant little Wales will not deserve its name if it leave the Carmarthenshire Infirmary long in this plight.

An American organist is reported to be changing colour in a disagreeable manner; once white, he has now become dark yellow, with the colour deepening to black at the joints. The doctors say the change is due to black jaundice and has no political signification. Still, it is not always physical disease which causes people to change colour.

---

Mr. C. S. Loch, the able secretary of the Charity Organization Society, writes from 15, Buckingham Street, W.C. : As your readers know, the Government have expressed their readiness to appoint a Select Committee of the House of Lords, to make inquiry with regard to all hospitals and dispensaries and charitable institutions within the metropolitan area for the care and treatment of the sick poor, which possess real property or invested personal property in the nature of an endowment of a permanent or temporary character, and with regard to the amount of accommodation provided out of the rates and the management thereof. A large number of the members of the profession signed the petition which has led to this investigation. I am desired to request you to allow me, through your columns, to ask your readers, especially those who are engaged in general practice, if they would furnish, for submission to the Select Committee, evidence in regard to such defects of our existing charitable medical system as have come to their knowledge, and, more particularly, explicit evidence in regard to cases in which persons have, without justification, claimed on charitable grounds the honorary services of the medical or surgical staff at hospitals and dispensaries when they were in a position to pay the ordinary fees. It would be of service, also, if they would give evidence in regard to cases in which the ailments were of such a trivial character as to make attendance at a hospital or dispensary unnecessary. I should add, perhaps, that a circular on this subject is being sent to the members of the profession who signed the petition. Communications in regard to this letter should be sent to me.

---

The Home Hospitals Association held their twelfth annual meeting at the Home Hospital, Fitzroy House, Fitzroy Square, W., on April 15th, Mr. Henry C. Burdett, vice-president, in the chair. Amongst those present were: Dr. Hare, Dr. Tom Robinson, Mr. Frederick Cox, Mr. Ruthven Pym, and Mr. W. E. Wallace. The report for the year stated that out of 514 applications 301 had been admitted. The average number of patients during the year was 17·5, and the average length of stay of each patient 18·2 days. The establishment of the Nurses' Home at 18, Fitzroy Square, had proved a success, there being a balance of £87 7s. 8d. left in hand on the year's accounts. The accounts for 1889, too, showed a satisfactory balance, the receipts being some £125 in excess of the expenditure. The chairman, in the course of his opening address, showed the influence the establishment of that Home Hospital had had upon the hospital system generally, and noted with satisfaction the excellent results that had been achieved by it during the past year, more patients having been relieved there than during any previous year. Speaking of nursing, the chairman said it was a popular error to suppose, as he believed was imagined by some people, that the public in employing trained nurses had no guarantee as to the character of the women they were introducing into their homes. This was not so, for every trained nurse was trained at a training school of repute, and these schools gave their nurses a certificate, which could be produced to every employer who required its production. If, then, they had any cause to complain, a letter addressed to the matron of the institution at which the nurse was trained would result in the reprimand, and, if necessary, the withdrawal of the certificate of that nurse. The officers and committee were re-elected, and votes of thanks were passed to the Medical Board of Reference, Miss Young (the lady superintendent), the executive staff, and the chairman.

